# Sociodemographic factors associated with the consumption of ultra-processed foods in Colombia

**DOI:** 10.11606/s1518-8787.2020054001176

**Published:** 2020-02-05

**Authors:** Neha Khandpur, Gustavo Cediel, Daniel Ayala Obando, Patrícia Constante Jaime, Diana C. Parra

**Affiliations:** I University of São PauloFaculty of Public HealthDepartment of NutritionSão PauloSPBrazilUniversity of São Paulo. Faculty of Public Health. Department of Nutrition. Center for Epidemiological Research in Nutrition and Health. São Paulo, SP, Brazil; II Harvard UniversityHarvard T.H. Chan School of Public HealthDepartment of NutritionCambridgeMAUSAHarvard University. Harvard T.H. Chan School of Public Health. Department of Nutrition. Cambridge, MA, USA; III University of AntioquiaSchool of Nutrition and DieteticsMedellínANTColombiaUniversity of Antioquia. School of Nutrition and Dietetics. Medellín, ANT, Colombia; IV Catholic University of ChileDepartment of StatisticsSantiagoRMChileCatholic University of Chile. Department of Statistics. Santiago, RM, Chile; V Washington University in St. LouisSchool of MedicineProgram in PhysiotherapySt. LouisMOUSAWashington University in St. Louis. School of Medicine. Program in Physiotherapy. St. Louis, MO, USA

**Keywords:** Food Consumption, Ultra-processed Foods, Socioeconomic Factors, Colombia, Diet Surveys

## Abstract

**OBJECTIVE:**

To analyze the consumption of ultra-processed foods in the Colombian population across sociodemographic factors.

**METHODS:**

We used data from the 2005 National Survey of the Nutritional Status in Colombia. Food consumption was assessed using a 24-hour food recall in 38,643 individuals. The food items were classified according to the degree and extent of industrial processing using the NOVA classification.

**RESULTS:**

The mean calorie contribution of ultra-processed foods ranged from 0.2% in the lowest quintile of consumers to 41.1% in the highest quintile of consumers. The greatest increases were due to the consumption of industrialized breads, sweet and savory snacks, sugary drinks, processed meats, and confectionery. No major differences were found in the consumption of ultra-processed foods between men and women. We observed significant differences by age, socioeconomic status, area of residence, and geographic region. Children and adolescents showed a higher intake of ultra-processed foods, almost double that of participants over 50 years of age. Children consumed significantly more snacks, confectionery products, processed cereals, milk-based drinks and desserts. Participants over 50 years consumed fewer products from these sub-groups of ultra-processed foods but had the highest consumption of industrialized bread. Individuals from urban areas, those with high socioeconomic status, participants residing in the Bogotá region had 1.5 to 1.7 times higher calorie intake from ultra-processed foods compared with those from a lower socioeconomic status and those residing in rural regions.

**CONCLUSION:**

In Colombia, industrialized bread is the ultra-processed product that is most easily assimilated into the traditional diet, along with snacks and sugary drinks. Children and adolescents residing in urban areas and households with greater purchasing power have some of the highest intakes of ultra-processed foods in the country.

## INTRODUCTION

Ultra-processed foods are industrial formulations made from substances derived from whole foods. Generally, they contain little or no natural foods, and have a high content of fat, salt, or/and sugar, and a low content of dietary fiber, proteins, micronutrients, and bioactive compounds. Examples of ultra-processed foods include snacks like industrialized ice cream, sugary drinks, chocolates, confectionery, french fries, hamburgers, and hot dogs^1,2^. These products are characterized by being ready to consume, hyperpalatable, extensively marketed, and having a long shelf life^1^.

Ultra-processed foods reflect a dietary pattern associated with a high content of nutrients related to chronic diseases^2^. These products dominate the food environment in developed countries, contributing almost 60.0% of the total energy consumed in the United States^6^, and 50.0% in Canada^7^. The high consumption of ultra-processed foods and the evidence showing an association with adverse health outcomes, such as obesity, metabolic syndrome, and breast cancer^8,9^, have resulted in efforts to reduce the intake of these products through regulation ^10,11^.

Ultra-processed foods contributed an average of 16.0% of the total daily calories in Colombia in 2005^5^. However, the combination of market forces, the increase in confidence to invest in the country following the end of the armed conflict, and the increase in purchasing power suggest an accelerated growth in the purchase and consumption of ultra-processed foods in the country^12^. Colombia is experiencing a nutritional, economic, and demographic transition, characterized by the decrease in the consumption of traditional meals, and an increase in urbanization and income, influenced by changing structures in the labor market^13,14^. Changes in market regulation in Colombia and the introduction of free trade agreements with countries that export ultra-processed foods ensure an increasing supply of these products^12^. Euromonitor International data show an annual percentage growth of more than 6.0% in the sales of all types of ultra-processed foods in Colombia, including frozen foods, sweet and savory snacks, canned/preserved foods, and dairy products^15^. The relative growth in sales of such product categories was higher in developing countries including Colombia than in industrialized countries between 1998 and 2012. Data from other Latin American countries such as Brazil, Chile, and Mexico have shown a substantial incorporation of ultra-processed foods in the diets of their populations ranging from 21.5% in 2008–2009, 28.6% in 2010, and 30% in 2012, respectively ^3,16,17^. These trends suggest that Colombia’s population, like it’s neighboring countries, is vulnerable to a very significant increase in the consumption of ultra-processed foods.

There has been no prior evaluation of the types of ultra-processed foods most consumed in Colombia. In this context of increasing foreign investment of food companies, expanding the diversity of ultra-processed foods on offer, and the growing trends of chronic diseases, it is important to assess the consumption pattern of these products. The study aimed to analyze the consumption of ultra-processed foods in the Colombian population, across sociodemographic factors.

## METHODS

This study used data from the first National Survey of the Nutritional Status of Colombia (ENSIN, 2005) and the National Survey of Demography and Health of Colombia (ENDS, 2005), conducted between October 2004 and July 2005 by the Colombian Institute of Family Welfare (ICBF) and Profamilia. The survey used a stratified, multi-stage, and cross-sectional design to obtain national and subregional representativeness (16 subregions), with over-sampling of rural areas and low socioeconomic (SES) groups. It included 99.0% of the urban and rural population with a response rate of 74.0%. The ENSIN sample size and sample design aimed to estimate proportion and prevalence^18^.

The dietary data were obtained from participants aged between 2 – 64 years using a 24-hour food recall. The 24-hour recall captured data from random days of the week including weekends. The interviewers used 60 standardized plastic models and photographs to improve the accuracy of the quantity and weight of the food and beverages consumed. They obtained information about the type of food, the name of the preparation, the ingredients, and the amount consumed. The person responsible for preparing the food was present at the time of the interview. In cases in which the food consumed by a child was in school or in a daycare center, the interviewer visited the school to obtain detailed information about the preparations. The quality of the data was monitored throughout the process, and the interview was repeated in case of inconsistencies^18^.

A total of 1,053 foods were classified into one of the four NOVA categories by the authors of this study. The categories are mutually exclusive and vary according to their extent and purpose of processing. They include unprocessed or minimally processed foods, processed culinary ingredients, processed foods, and ultra-processed foods^1^. The foods were categorized into one of 33 subgroups. In 5.6% of the cases, it was not possible to disaggregate the typical culinary dishes into their constituent ingredients (for example, “Lasagna,” “Corn kernel cakes,” “Tamale”), and these products were classified as minimally processed foods (“freshly prepared food” ). The intake of energy and nutrients for each food at an individual level was calculated using software developed by the School of Nutrition and Dietetics at the University of Antioquia, Medellín, Colombia^18^.

This study used self-reported data on sex (male/female), age (2 to 9, 10 to 19, 20 to 34, 35 to 49, and ≥ 50 years), SES, and geographic regions compiled by the ENDS. The SES of the sample was evaluated using the SISBEN composite index. This is a family welfare index composed of 24 variables across four dimensions: health, education, housing, and vulnerability, and it includes indicators such as social security, years of schooling, employment, and income *per capita*^19^. SISBEN level 1 indicates low SES, while “Level 4 and higher” indicates high SES. The area of residence indicator comprised of urban, central, and rural categories. The geographical regions included Atlantic, Eastern, Central, Pacific, Bogota, Orinoquía, and the Amazon.

The ENSIN data were combined with the ENDS data to link food consumption with demographic information at the individual level. Responses with missing information for total energy, with extreme total energy intakes (< 200 kcal and > 5,000 kcal)^5^, and responses from pregnant women were excluded from the analysis. The final sample size included 38,643 individuals. Sample weights were used in all analyses to consider the differential probabilities of selection. Individuals were classified into quintiles according to the dietary contribution of the ultra-processed foods (% of total energy intake). We analyzed the relative energy consumption (% of total energy intake) for the four NOVA categories and subgroups across quintiles. Gross and adjusted linear regression analyses were performed with sociodemographic indicators and all NOVA categories and subgroups of ultra-processed foods. The significance level was established as an alpha of 5.0% and a p-value of 0.05. Visual diagnostic tests for the ordinary least squares (OLS) regression suggested a fairly normal distribution of errors, and slight deviations from normality in the upper and lower tails. Data analyses were performed in STATA 14, and the consumption map was designed in R version 3,4,4.

## RESULTS

The participants’ mean age was 26.5 years [standard deviation (SD) = 0.2], 51.9% of the sample was composed of women, 29.4% represented low SES, and 5.1% represented high SES. Three quarters of the population resided in urban areas, and the highest number of participants were from the Atlantic (25.3%) and Central (23.8%) regions (Table 1). Natural or minimally processed foods provided 63.3% of the total energy consumed by the sample, while the energy from processed culinary ingredients, processed foods, and ultra-processed foods accounted for 15.8%, 4.9% and 15.9%, respectively. Bananas, roots and tubers were the most consumed subcategory among minimally processed foods. Table sugar, cheese and industrialized breads were the most consumed culinary ingredients, processed foods, and ultra-processed foods, respectively (Table 2).

**Table 1:** Demographic characteristics. National survey of the nutritional status of the Colombian population. Colombia, 2005.

Indicator	Distribution	

	Mean	Standard error
Age (years)	26.5	0.2
	n	%^*^
Sex		
Female	19,991	51.9
Male	18,527	48.1
Age groups (years)		
2–9	7,126	18.5
10–19	8,859	23.0
20–34	10,130	26.3
35–49	7,703	20.0
≥ 50	4,699	12.2
Socioeconomic status		
Level 1 (low)	10,169	29.4
Level 2	14,213	36.9
Level 3	10,977	28.5
Level 4 (high)	1,964	5.1
Area of residence		
Urban	28,272	73.4
Central	5,854	15.2
Rural	4,391	11.4
Region		
Atlantic	9,745	25.3
Eastern	6,471	16.8
Central	9,167	23.8
Pacific	6,509	16.9
Bogotá	6,124	15.9
Orinoquía and Amazon	423	1.1

* Weighted percentages, may not add up to 100 due to rounding

Population size with sample weights = 38,519,068

**Table 2:** Distribution (%) of total energy intake according to the NOVA food classification using the quintiles (Q) of the percentage of energy from ultra-processed foods. Colombia, 2005

Food Groups	Total 1,835 kcal	Q1 1,511 kcal	Q2 1,879 kcal	Q3 1,873 kcal	Q4 1,889 kcal	Q5 2,039 kcal
UPF calorie percentage		0–1.5%	1.6–9.3%	9.4–17.2%	17.3–28.7%	28.8–100%

**Natural or minimally processed foods**	**63.3**	**76.1**	**70.7**	**64.9**	**58.4**	**44.1**
Bananas, roots and tubers (includes flours)	9.0	22.4	18.4	15.8	13.4	9.3
Cereals, grains (includes flours)	14.2	19.4	15.8	14.3	12.0	9.0
Culinary preparations (ready to eat)^a^	7.1	7.6	8.9	7.4	6.6	4.8
Milk, yogurt (natural)	5.5	3.9	5.2	6.1	6.7	5.8
Red meat	5.1	5.3	5.4	5.4	4.9	4.0
Fruits^b^	3.6	4.4	4.1	3.7	3.2	2.4
Beans, pulse, legumes (includes flours)	3.5	4.2	4.0	3.7	3.3	2.0
Eggs	2.5	2.3	2.7	2.6	2.7	2.2
Poultry meat	2.2	1.9	2.2	2.4	2.5	2.0
Vegetables	1.6	1.5	1.7	1.7	1.7	1.4
Seafood	0.8	1.7	0.8	0.7	0.4	0.3
Natural fruit juice	0.3	0.2	0.2	0.3	0.3	0.3
Other minimally processed foods^c^	0.9	1.4	1.1	0.9	0.7	0.5
**Processed culinary ingredients**	**15.8**	**19.3**	**18.3**	**16.3**	**14.1**	**10.4**
Sugar	8.9	11.2	10.6	9.3	7.7	5.3
Vegetable oils	6.1	6.9	6.7	6.2	5.8	4.6
Animal fat	0.8	1.2	1.0	0.8	0.6	0.4
Other culinary ingredients^c^	0.0	0.0	0.0	0.0	0.0	0.01
**Groups 1 + 2**	**79.1**	**95.4**	**89.0**	**81.2**	**72.5**	**54.6**
**Processed foods**	**4.9**	**4.4**	**5.4**	**5.6**	**5.0**	**4.4**
Cheeses	1.9	2.2	2.0	2.1	1.9	1.7
Bakery (fresh unpackaged)	1.7	1.0	1.9	2.1	2.0	1.4
Meats (canned, smoked)	0.2	0.2	0.3	0.3	0.2	0.2
Canned fruits and vegetables	0.1	0.0	0.1	0.1	0.1	0.1
Wine and beer	0.1	0.0	0.1	0.1	0.1	0.1
Other processed foods^c^	0.0	0.0	0.0	0.0	0.0	0.01
**Ultra-processed foods**	**15.9**	**0.2**	**5.6**	**13.2**	**22.5**	**41.1**
Industrialized breads	5.0	0.0	2.1	5.3	8.2	10.3
Snacks (sweet and savory)^d^	2.5	0.0	0.7	1.8	3.2	7.5
Sugary drinks^e^	2.5	0.0	0.1	2.2	3.7	6.0
Confectionery (chocolate, candies, sweets)	1.5	0.0	0.8	1.3	2.2	3.6
Processed meats	1.3	0.0	0.3	0.9	1.9	3.9
Ready-to-eat preparations “junk food”^f^	0.6	0.0	0.0	0.1	0.4	2.8
Commercial desserts	0.5	0.0	0.1	0.3	0.7	1.5
Industrial breakfast cereals	0.3	0.0	0.1	0.1	0.4	0.9
Industrial dairy drinks^g^	0.2	0.0	0.1	0.2	0.3	0.5
Other ultra-processed foods^c^	1.4	0.0	0.0	1.1	1.5	4.2

^a^ Includes sweet or savory culinary preparations that cannot be disaggregated into their ingredients (a combination of ingredients mainly from group 1: pastries, donuts, pies, etc.).

^b^ Includes fruit pulps and coconut water.

^c^ Other: Natural or minimally processed foods: cocoa, insect meat, coconut milk, soy milk, nuts, coffee, tea and tofu. Processed culinary ingredients: table salt, pepper, vinegar, yeast, vanilla extract, unflavored gelatin. Processed foods: salted, sweetened, or oil-added nuts and seeds, condensed milk. Ultra-processed foods: margarines, broth tablets, sauces, commercial baby foods, distilled alcohols.

^d^ Includes mix of snacks, industrial crackers and cookies, wafers.

^e^ Includes industrial fruit juices.

^f^ Includes frozen pizza, packaged soups, pre-cooked pasta.

^g^ Includes custard, flavored and malted yogurts.

The percentage contribution of total calories from natural or minimally processed foods, and culinary ingredients and their subgroups, decreased across the quintiles of consumption of ultra-processed foods. Calories from natural or minimally processed foods decreased by 32.0% between the first quintile (76.1%) and last quintile (44.1%). The consumption of natural yogurt increased between the quintiles, while the consumption of vegetables was not very high (1.5%) and remained unchanged between the quintiles. The percentage of calories from all processed foods remained relatively unchanged, increasing marginally between quintiles 2 and 3, before decreasing again.

The mean calorie contribution of ultra-processed foods ranged from 0.2% in the first quintile to 41.1% in the last quintile. The greatest increases came from the consumption of industrialized breads, followed by sweet and savory snacks, sugary drinks, processed meats, and confectionery products. Ready-to-eat foods, desserts, industrialized breakfast cereals, and dairy drinks also increased between quintiles, but to a lesser degree.

**Figure 1:**
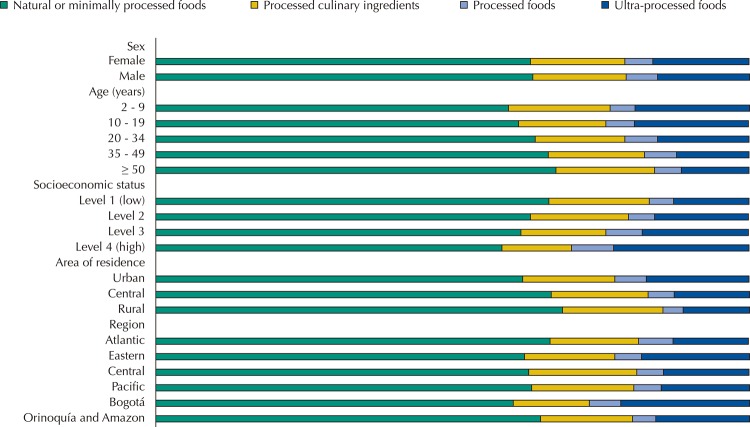
Distribution of total energy intake according to NOVA classification and sociodemographic determinants. Colombia, 2005.

**Figure 2:**
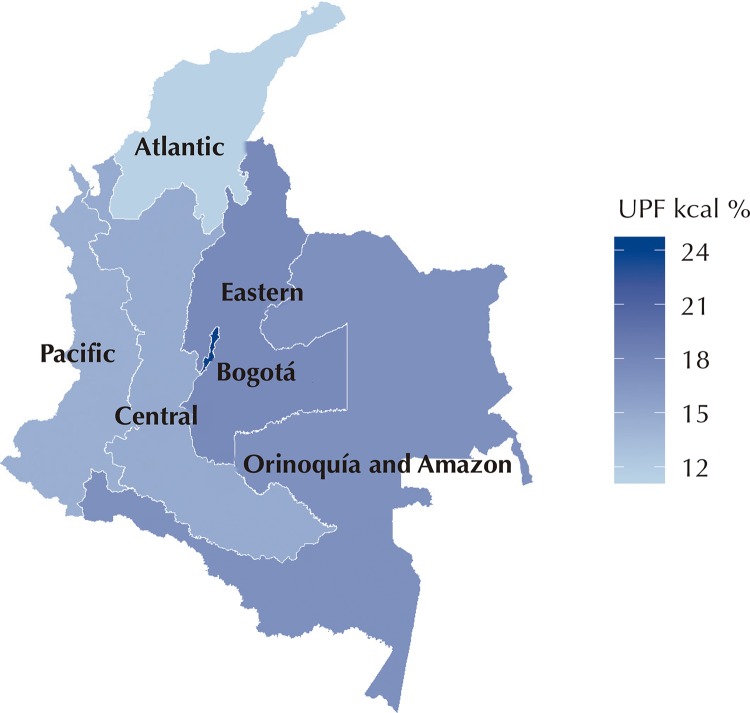
Distribution of ultra-processed foods (UPF) consumption in the regions. Colombia, 2005.

No major differences were found in the consumption of ultra-processed foods between men and women. We observed significant differences by age, SES, area of residence, and geographic region. The oldest participants had the highest consumption of natural or minimally processed foods, while the youngest participants (2–9 years old) had the highest intake of ultra-processed foods: almost double that of the participants aged over 50 years. The highest consumers of natural or minimally processed foods and the lowest consumers of ultra-processed foods were from the lowest SES, resided in rural areas, or in the Atlantic region. Individuals from the urban area, with high SES, residing in the Bogotá region had 1.5 to 1.7 times higher calorie intake from ultra-processed foods compared to those with low SES and individuals residing in rural areas (Figures 1 and 2 and Table 3).

**Table 3:** Variation in the mean consumption of ultra-processed foods (UPF) by the sociodemographic determinants in the population. Colombia, 2005.

Sociodemographic factors	UPF energy % (SE)	Beta (not adjusted)	p	Adjusted UPF energy % (SE)	Beta (adjusted)	p
Sex						
Female	16.2 (0.3)	Reference	-	16.2 (0.2)	Reference	-
Male	15.5 (0.4)	-0.7	0.012	15.5 (0.2)	-0.6	0.007
Age (years)						
2–9	18.5 (0.4)	Reference	-	19.3 (0.3)	Reference	-
10–19	18.6 (0.4)	0.1	0.678	19.3 (0.2)	-0.1	0.718
20–34	16.0 (0.4)	-2.5	< 0.001	15.4 (0.3)	-3.9	< 0.001
35–49	12.6 (0.4)	-5.9	< 0.001	12.2 (0.3)	-7.1	< 0.001
≥ 50	11.8 (05)	-6.8	< 0.001	11.4 (0.4)	-7.9	< 0.001
Socioeconomic status						
Level 1 (low)	10.4 (0.4)	Reference		12.7 (0.3)	Reference	-
Level 2	16.5 (0.4)	6.1	< 0.001	15.8 (0.3)	3.1	< 0.001
Level 3	19.4 (0.4)	9.0	< 0.001	17.9 (0.3)	5.2	< 0.001
Level 4 (high)	23.6 (1.1)	13.2	< 0.001	22.8 (1.0)	10.1	< 0.001
Area of residence						
Urban	18.2 (0.3)	Reference		17.3 (0.2)	Reference	-
Central	9.2 (0.7)	-8.9	< 0.001	12.6 (0.5)	-4.6	< 0.001
Rural	10.4 (0.7)	-7.8	< 0.001	11.2 (0.6)	-6.1	< 0.001
Region						
Atlantic	11.4 (0.6)	Reference	-	12.7 (0.3)	Reference	-
Eastern	17.6 (0.8)	6.2	< 0.001	18.1 (0.4)	5.4	< 0.001
Central	14.8 (0.7)	3.5	< 0.001	14.4 (0.4)	1.7	0.001
Pacific	14.4 (0.6)	3.1	< 0.001	14.9 (0.4)	2.2	< 0.001
Bogotá	24.4 (0.6)	13.0	< 0.001	21.6 (0.5)	8.9	< 0.001
Orinoquía and Amazon	17.1 (0.6)	5.7	< 0.001	15.7 (0.6)	3.0	< 0.001

SE = Standard Error

Women consumed more breads, snacks and confectionery products while men consumed more sugary drinks and ready-to-eat meals. Children aged below 9 years consumed significantly more snacks, confectionery products, processed cereals, dairy drinks, and desserts. Participants older than 50 years consumed fewer products from these subgroups but had the highest consumption of industrialized bread. As for sugary drinks, a different trend was also observed. People aged 20–34 years had the highest intakes (3.3% of the total energy of sugary drinks) compared with the older adults (1.3%) and children (2.1%). Similarly, we observed higher intake of ultra-processed foods in the high SES class for most ultra-processed foods such as snacks, sugary drinks, processed meat, ready-to-eat foods, and desserts. These consumers had an intake comparable to that of those with low SES in processed bread and confectionery (Table 4).

**Table 4:** Percentage of energy from ultra-processed foods (UPF) and their subgroups according to sociodemographic determinants. Colombia, 2005.

Indicators	All UPF	Industrialized breads	Snacks (sweet and savory)^b^	Sugary drinks^c^	Confectionery	meats	Junk food^d^	Commercial desserts	Cereals	Dairy Drinks^e^	Others UPF^f^
Sex											
Female	16.2 (0.2)	5.2 (0.1)	2.8 (0.1)	2.2 (0.1)	1.7 (0.1)	1.4 (0.1)	0.6 (0.0)	0.6 (0.0)	0.4 (0.0)	0.2 (0.0)	1.2 (0.1)
Male	15.5 (0.2)	4.8 (0.1)	2.2 (0.1)	2.8 (0.1)	1.3 (0.0)	1.3 (0.1)^a^	0.7 (0.1)	0.4 (0.0)	0.2 (0.0)	0.2 (0.0)^ a^	1.6 (0.1)
Age (years)											
2 – 9	19.3 (0.3)	5.2 (0.1)	4.5 (0.1)	2.1 (0.1)	2.3 (0.1)	1.6 (0.1)	0.5 (0.1)	0.9 (0.1)	0.6 (0.0)	0.5 (0.0)	0.9 (0.1)
10 – 19	19.3 (0.2)	5.4 (0.1)^ a^	3.8 (0.1)	2.8 (0.1)	2.1 (0.1)	1.7 (0.1)^ a^	0.9 (0.1)	0.7 (0.0)	0.3 (0.0)	0.2 (0.0)	1.2 (0.1)
20 – 34	15.4 (0.3)	4.6 (0.2)	1.8 (0.1)	3.3 (0.1)	1.1 (0.1)	1.4 (0.1)^ a^	0.6 (0.1)^ a^	0.3 (0.0)	0.2 (0.0)	0.1 (0.0)	1.9 (0.2)
35 – 49	12.2 (0.3)	4.7 (0.2)	1.1 (0.1)	2.1 (0.1)^ a^	1.0 (0.1)	1.0 (0.1)	0.3 (0.1)	0.3 (0.1)	0.1 (0.0)	0.1 (0.0)	1.4 (0.1)
≥ 50	11.4 (0.4)	5.5 (0.3)^ a^	0.9 (0.1)	1.3 (0.1)	1.0 (0.1)	0.8 (0.1)	0.4 (0.1)^ a^	0.2 (0.1)	0.1 (0.0)	0.1 (0.0)	1.3 (0.2)^ a^
Socioeconomic status											
Level 1 (low)	12.7 (0.3)	4.4 (0.2)	1.9 (0.1)	1.8 (0.1)	1.4 (0.1)	1.0 (0.1)	0.3 (0.0)	0.3 (0.0)	0.1 (0.0)	0.1 (0.0)	1.3 (0.1)
Level 2	15.8 (0.3)	5.4 (0.1)	2.3 (0.1)	2.5 (0.1)	1.6 (0.1)	1.3 (0.1)^ a^	0.6 (0.0)	0.5 (0.0)	0.3 (0.0)	0.2 (0.0)	1.3 (0.1)^ a^
Level 3	17.9 (0.3)	5.2 (0.1)	3.1 (0.1)	2.9 (0.1)	1.5 (0.1)^ a^	1.6 (0.1)	0.8 (0.1)	0.6 (0.1)	0.4 (0.1)	0.3 (0.0)	1.5 (0.1)^ a^
Level 4 (high)	22.8 (1.0)	5.2 (0.4)^ a^	3.8 (0.4)	3.7 (0.3)	1.4 (0.2)^ a^	2.3 (0.4)	1.8 (0.3)	1.0 (0.1)	0.7 (0.1)	0.6 (0.1)	2.2 (0.4)
Area of residence											
Urban	17.3 (0.2)	5.4 (0.1)	2.6 (0.1)	2.8 (0.1)	1.5 (0.1)	1.5 (0.1)	0.7 (0.0)	0.6 (0.0)	0.3 (0.0)	0.2 (0.0)	1.4 (0.1)
Central	12.6 (0.5)	4.2 (0.2)	2.3 (0.2)	1.8 (0.1)	1.5 (0.1)^ a^	0.9 (0.1)	0.4 (0.0)	0.4 (0.1)^ a^	0.2 (0.0)	0.1 (0.0)	0.8 (0.1)
Rural	11.2 (0.6)	3.5 (0.3)	1.5 (0.1)	1.4 (0.2)	1.3 (0.2)^ a^	0.7 (0.2)	0.2 (0.1)	0.3 (0.1)^ a^	0.1 (0.0)	0.1 (0.0)	2.1 (0.4)^ a^
Region											
Atlantic	12.7 (0.3)	3.5 (0.1)	2.2 (0.1)	2.4 (0.1)	0.5 (0.0)	1.3 (0.1)	0.5 (0.0)	0.3 (0.0)	0.2 (0.0)	0.2 (0.0)	1.4 (0.1)
Eastern	18.1 (0.4)	6.3 (0.3)	2.4 (0.1)^ a^	3.0 (0.1)	2.1 (0.1)	1.1 (0.1)^ a^	0.6 (0.1)^ a^	0.6 (0.1)	0.4 (0.1)	0.1 (0.0)^ a^	1.4 (0.1)^ a^
Central	14.4 (0.4)	3.1 (0.2)^ a^	2.6 (0.1)	2.3 (0.1)^ a^	1.3 (0.1)	1.7 (0.1)	0.7 (0.1)	0.6 (0.1)	0.3 (0.0)	0.3 (0.0)	1.5 (0.2)^ a^
Pacific	14.9 (0.4)	5.5 (0.2)	2.6 (0.1)	2.1 (0.1)	0.9 (0.1)	1.4 (0.1)^ a^	0.4 (0.1)^ a^	0.4 (0.0)	0.2 (0.0)^ a^	0.2 (0.0)^ a^	1.1 (0.1)^ a^
Bogotá	21.6 (0.5)	8.2 (0.3)	2.8 (0.2)	2.6 (0.2)^ a^	3.3 (0.2)	1.0 (0.1)^ a^	0.9 (0.1)	0.7 (0.1)	0.4 (0.1)	0.2 (0.0)^ a^	1.4 (0.2)^ a^
Orinoquía and Amazon	15.7 (0.6)	4.9 (0.3)	2.5 (0.2)^ a^	3.4 (0.2)	1.7 (0.2)	1.1 (0.1)^ a^	0.6 (0.1)^ a^	0.4 (0.1)^ a^	0.3 (0.0)	0.1 (0.0)^ a^	0,7 (0,1)

^a^ There are no significant differences compared with the reference group in each variable (sex: female, age: 2–9 years, region: Atlantic, Area: urban, Socioeconomic status: Low, after adjusting for all sociodemographic variables.

^b^ Includes mix of snacks, industrial crackers and cookies, wafers.

^c^ Includes industrial fruit juices.

^d^ Includes frozen pizza, packaged soups, pre-cooked pasta.

^e^ Includes custard, flavored and malted yogurts.

^f ^Includes margarines, broth tablets, sauces, commercial baby meals, distilled alcohols.

Participants that resided in urban areas consumed more ultra-processed foods. However, their consumption was not significantly different compared with residents in rural regions, with regards to confectionery sweets. We observed similar trends among residents of the Bogotá region who consumed significantly more industrialized bread, confectionery, snacks, ready-to-eat foods, and desserts compared to other regions. However, the consumption of processed meats was the lowest. The residents of Orinoquía and the Amazon had the highest intake of sugary drinks, while those of the Central region exceeded the consumption of processed meat. Almost no differences were observed between geographic regions for the intake of dairy drinks.

## DISCUSSION

The data showed a wide range in the consumption patterns of ultra-processed foods in Colombia across various sociodemographic factors. Those individuals with the lowest consumption of ultra-processed foods (first quintile of consumption of ultra-processed foods, < 1% of total energy consumption) obtained more than 95% of their energy from natural or minimally processed foods and culinary ingredients. On the other hand, individuals with higher consumption of ultra-processed foods replaced almost half of the calories from natural or minimally processed foods – they obtained 41% of their energy consumption from ultra-processed foods and 44% from natural or minimally processed foods. Industrialized bread was the largest contributor to total energy from ultra-processed foods. Adolescents and urban residents were the largest consumers of ultra-processed foods, especially snacks, processed cereals, dairy drinks, and junk food. Men consumed more sugary drinks and ready-to-eat meals.

As the total consumption of ultra-processed foods increased, industrialized bread, snacks, and sugary drinks provided the greatest amount of energy, surpassing the consumption of bananas, roots and tubers, and cereals from the group of natural or minimally processed foods. The subgroups of ultra-processed foods that supply most calories vary among other countries in South America. Desserts, cookies, cakes, and sugary drinks are the main contributors to energy in Mexico and Chile^16,20^. Desserts, fast foods, and sugary drinks are the main contributors in Brazil^4^. Confectionery, processed meats, and ready-to-eat meals also increased in Colombia, but to a lesser extent. In general, the consumption of ultra-processed foods in Colombia remains relatively low, but with enormous potential for growth in all subgroups according to recent purchase trend data^15^.

In Colombia, people aged below 19 years living in the capital Bogotá, and with a high SES were the main consumers of ultra-processed foods. Of these, children were the most vulnerable group for the consumption of these products. This is particularly worrying considering the long-term health consequences of eating ultra-processed foods^8,9^. A dietary pattern characterized by the consumption of subgroups of ultra-processed foods such as hamburgers and hot dogs was associated with overweight among Colombian children aged 5–12 years, particularly those in the high SES^21^. Rural residents and older adults are likely to have more traditional cooking and eating practices and more stable dietary patterns. These groups may also be more resistant to marketing practices that appeal to the younger generation, with less stable dietary patterns, and therefore more likely to try these products.

Aspects such as exposure, availability, access, and penetration of these ultra-processed foods in Colombia’s food environment may also explain the highest consumption of these products in urban areas, high SES areas, and in large cities (mainly in the capital, Bogotá). These segments may have greater exposure to marketing, greater purchasing power to access these products, and a wider palate to adapt to new tastes. Colombians residing in rural areas, in the Atlantic, Central, and Pacific regions, or those in the lower SES seem protected from exposure to these ultra-processed foods, while retaining traditional culinary preparations. This is probably due to lower exposure to the commercial marketing of these products and to a lower disposable income.

The Colombian consumption pattern of ultra-processed foods among children and adolescents reflects the global pattern: the United Kingdom, the United States, Canada, Chile, France, and Mexico show a higher consumption of ultra-processed foods in these age groups^7,16,20^. On the other hand, consumption is not as consistent by SES. In Mexico and Brazil, as in Colombia, the richest and most educated consume more ultra-processed foods, with a dose-response in relation to the increase according to SES^20,24^. Chile also showed higher consumption in those with higher incomes, but no differences were found by education^16^. However, the consumption of ultra-processed foods was higher among those with lowest education and income level in the United Kingdom, France, and the United States. These results indicate a distinction in consumption patterns according to the nutritional transition stage of each country. In developing countries in the intermediate stage (patterns 3 and 4) of nutritional transition, the purchase and consumption of ultra-processed foods were boosted by individuals with high income levels. These countries move away from the intake of starchy foods (corn, wheat, rice, oats, etc.) and protein-energy deficiencies towards noncommunicable diseases^25^. The opposite was observed in developed countries that are nearing the end (pattern 4) of nutritional transition. In these countries, consumption was relatively low among the richest, however, their percentage of daily energy from ultra-processed foods was greater than 50%^25^.

Few studies have examined the differences within the ultra-processed food subgroups. In Norway, men were more likely to be classified as large consumers of ultra-processed dinner products and fast foods, but not of snacks and sugary drinks, compared with women^26^. Those with a high level of education were less likely to consume ultra-processed foods at dinner and snacks and sugary drinks during the day compared with those with a low level of education. These results were different from those seen in this study and are probably explained by the later stage of the nutritional transition (pattern 4) in Norway. Among children, there is some consistency in the types of ultra-processed foods consumed. Analyses of the main components of food consumption in 12 countries show that ultra-processed foods such as fast foods, ice cream, fried foods, fries, and sugary drinks more strongly characterize unhealthy eating patterns among children aged 9–11 years^27^. Recent data from Colombia on the frequency of consumption of sugary drinks show that 85.3% of the children aged 5–17 years consume these drinks 0.71 time per day^28^. The percentage contribution of sugary drinks to daily energy was not available, but frequency trends indicate a very present and worrying consumption pattern among children and adolescents.

Regulation is necessary to protect nutritional status and well-being, especially of children and adolescents^12^. Strategies may include: (i) prohibiting the sale of ultra-processed food in schools and playgrounds; (ii) better disseminating health promotion tools, such as food-based dietary guidelines that consider the extent and purpose of the processing implemented in Brazil^29^ and Uruguay^30^; (iii) including appropriate warning labels and imposing initiatives to encourage healthier options at points of purchase as has been done in Chile^10^.

This study has its limitations. All dietary information was collected by 24-hour dietary recalls. In addition to the memory bias, participants may choose not to disclose information about certain food products that are considered socially undesirable, both in terms of product categories and quantities consumed. This single point of dietary information may not capture the participants’ usual diet and, therefore, be less representative of their intake. Although the analyses controlled for several sociodemographic variables, residual confusion is difficult to eliminate. However, the probabilistic nature of the sample studied, the national representativeness of the Colombian population, the use of the latest national survey with 24-hour dietary recall available, and the standardization of the collection of dietary data are some of the strengths of this study. It is probable that the consumption of ultra-processed foods in Colombia has changed since the publication of these data. This study provides reference intake levels and could serve as a baseline for comparison with more recent dietary assessments, when available, to capture trends in the consumption of ultra-processed foods in Colombia.

## CONCLUSION

This study characterizes the ultra-processed products that are consumed most frequently and that can be considered as the entry points of ultra-processed foods into the Colombian diet. Sociodemographic differences are explored to identify segments of the population that can be considered the most vulnerable to the consumption of these foods. Industrialized bread is the ultra-processed food that is most easily assimilated into the traditional diet, along with snacks and sugary drinks. Children and adolescents adapt more easily to these products and, therefore, are more vulnerable to their deleterious effects on health. In general, the consumption of ultra-processed foods is not as high in Colombia as in other countries, but has great potential to increase. These data also encourage a review of the current health promotion and prevention policy and regulatory efforts in Colombia related to the promotion and sale of these products, particularly with regards to vulnerable sub-groups such as children and adolescents.
