# Supplementary material for: Sociodemographic factors associated with the consumption of ultra-processed foods in Colombia
Source: Rev Saude Publica. 2020 Feb 5;54:19. doi: 10.11606/s1518-8787.2020054001176 (PMC7006913; doi:10.11606/s1518-8787.2020054001176)
Supplement: Translation

# Translation: Factores sociodemográficos asociados al consumo de alimentos ultraprocesados en Colombia

**Authors:** Neha Khandpur, Gustavo Cediel, Daniel Ayala Obando, Patrícia Constante Jaime, Diana C. Parra

**Affiliations:** I Brasil Universidade de São Paulo. Faculdade de Saúde Pública. Departamento de Nutrição. Núcleo de Pesquisas Epidemiológicas em Nutrição e Saúde. São Paulo, SP, Brasil; II USA Harvard University. Harvard T.H. Chan School of Public Health. Departamento de Nutrición. Cambridge, MA, USA; III ColombiaUniversidad de Antioquia. Escuela de Nutrición y Dietética. Medellín, ANT, Colombia; IV Chile Pontificia Universidad Católica de Chile. Departamento de estadística. Santiago, RM, Chile; V USAWashington University in St. Louis. School of Medicine. Program in Physiotherapy. St. Louis, MO, USA

**Keywords:** Consumo de Alimentos, Factores Socioeconómicos, Inquéritos sobre Dietas, Alimentos Ultraprocesados

## Abstract

**OBJETIVO:**

Analizar el consumo de alimentos ultraprocessados en la población colombiana según factores sociodemográficos.

**MÉTODOS:**

Se usaron datos de la Encuesta Nacional de la Situación Nutricional en Colombia del año 2005. El consumo de alimentos se evaluó por medio de recordatorio 24 horas en 38.643 individuos. Los ítems alimentarios se clasificaron según el grado y extensión de procesamiento industrial usando la propuesta NOVA.

**RESULTADOS:**

La contribución promedio de calorías de los alimentos ultraprocesados varió del 0,2% en el primer quintil al 41,1% en el ultimo quintil. Los mayores incrementos se dieron por el consumo de panes industrializados, snacks dulces y salados, las bebidas azucaradas, las carnes procesadas y los productos de confitería. No hubo grandes diferencias en el consumo de alimentos ultraprocesados entre hombres y mujeres. Se observaron diferencias significativas por edad, estatus socioeconómico, área de residencia y región geográfica. Los niños y adolescentes presentaron mayor ingesta de alimentos ultraprocesados, casi el doble que los participantes mayores de 50 años. Los niños consumieron significativamente mayor cantidad de snacks, productos de confitería, cereales procesados, bebidas a base de leche y postres. Mientras que los participantes mayores de 50 años consumieron menor cantidad de productos de estos subgrupos de alimentos ultraprocesados, pero tenían el consumo más alto de pan industrializado. Los habitantes urbanos, con alto estatus socioeconómico, que residían en la región de Bogotá tenían entre 1,5 a 1,7 más veces de ingesta calórica de alimentos ultraprocesados en comparación con sus contrapartes de bajo estatus socioeconómico, y sus contrapartes rurales.

**CONCLUSIÓN:**

En Colombia, el pan industrializado es el alimento ultraprocesado más fácilmente asimilable en la dieta tradicional, junto con los snacks y las bebidas azucaradas. Los niños y adolescentes residentes en zonas urbanas y hogares con mayor poder adquisitivo fueron más vulnerables en el consumo de alimentos ultraprocesados.

## INTRODUCCIÓN

Los alimentos ultraprocesados son formulaciones industriales fabricadas de sustancias derivadas de alimentos o sintetizadas de otras fuentes orgánicas. Generalmente, contienen poco o nada de alimentos naturales, un alto contenido de grasas, sal o azúcar y bajo contenido de fibra dietética, proteínas, micronutrientes y compuestos bioactivos. Entre los ejemplos de alimentos ultraprocesados se incluyen: productos snacks con alto contenido de azúcar, grasa o sal, helados industrializados, bebidas azucaradas, chocolates, confitería, papas fritas, hamburguesas, y perros calientes^1,2^. Estos productos se caracterizan por ser listos para consumir, hiperpalatables, muy publicitados, y por tener una larga vida útil^1^.

Los alimentos ultraprocesados generan un patrón dietético asociado a un alto contenido de nutrientes relacionados con enfermedades crónicas^2^. Estos productos dominan el sistema alimentario en los países desarrollados, contribuyendo con casi el 60,0% de la energía total consumida en los Estados Unidos^6^, y con el 50,0% en Canadá^7^. El alto consumo de alimentos ultraprocesados, y la evidencia mostrando una asociación con resultados adversos para la salud, como el obesidad, síndrome metabólico y cáncer de mama^8,9^, han impulsado esfuerzos para reducir la ingesta de estos productos a través de la regulación y fiscalización^10,11^

Los alimentos ultraprocesados contribuyeron en promedio con un 16,0% del total de calorías diarias en Colombia en el año 2005^5^. Sin embargo, la combinación de las fuerzas del mercado, el aumento en la confianza para invertir en el país por el pacto del término del conflicto armado y el mejoramiento en el poder adquisitivo sugieren un aumento acelerado en la compra y el consumo de alimentos ultraprocesados en el país^12^. Colombia experimenta una transición nutricional, económica y demográfica, caracterizada por la disminución en el consumo de preparaciones tradicionales, el aumento de la urbanización y en los ingresos influenciado por estructuras cambiantes en el mercado laboral^13,14^. Los cambios en la regulación del mercado y la introducción de acuerdos de libre comercio con países exportadores de alimentos ultraprocesados aseguran un suministro cada vez mayor de estos productos^12^. Los datos de Euromonitor International muestran en Colombia un crecimiento porcentual anual de más del 6,0% en las ventas de todos los tipos de alimentos ultraprocesados, incluyendo los alimentos congelados, aperitivos dulces y salados, alimentos enlatados/conservados y productos lácteos^15^. El crecimiento relativo en las ventas dentro de tales categorías de productos fue mayor en países en desarrollo incluyendo Colombia que en los países industrializados entre 1998 y 2012. Otros países de América Latina como Brasil, Chile y México han encontrado una incorporación sustancial de los alimentos ultraprocesados en las dietas de sus poblaciones de 21,5% en 2008-2009, 28,6% en 2010 y 30% en 2012, respectivamente^3,16,17^. Estas tendencias sugieren que la población de Colombia, al igual que en países vecinos, es vulnerable a un aumento dramático en el consumo de alimentos ultraprocesados.

No existe un reporte previo de los tipos de alimentos ultraprocesados de mayor consumo en Colombia. En este contexto de aumento de la inversión extranjera de las empresas de fabricación de alimentos, ampliando la diversidad en alimentos ultraprocesados en oferta, y de las crecientes tendencias de las enfermedades crónicas, es importante conocer el patrón de consumo de esos productos. El objetivo del estudio fue analizar el consumo de alimentos ultraprocesados en la población colombiana, según factores sociodemográficos.

## MÉTODOS

Este estudio utilizó datos de la primera Encuesta Nacional de la Situación Nutricional de Colombia (ENSIN, 2005) y la Encuesta Nacional de Demografía y Salud de Colombia (ENDS, 2005), realizada entre octubre de 2004 y julio de 2005 por el Instituto Colombiano de Bienestar Familiar (ICBF) y Profamilia. La encuesta utilizó un diseño estratificado, de etapas múltiples y de corte transversal para obtener representatividad nacional y subregional (16 subregiones), con sobre-muestreo de áreas rurales y grupos de bajo estatus socioeconomico (ESE). Incluyó el 99,0% de la población urbana y rural con una tasa de respuesta del 74,0%. El tamaño de la muestra y el diseño muestral de la ENSIN fueron realizados para calcular los estimativos de proporción y de prevalencias, como también modelos estadísticos para evaluar asociaciones^18^.

Los datos dietéticos se obtuvieron utilizando un recordatorio de 24 horas, administrado por el entrevistador, en edades entre los 2 y 64 años para ambos sexos. El recordatorio de 24 horas capturó datos de días aleatorios en semana y fines de semana. Los entrevistadores utilizaron 60 modelos estandarizados de plástico y fotografías para mejorar la precisión de la cantidad y peso de los alimentos y bebidas consumidas. Se registró la información sobre el tipo de alimento, el nombre de la preparación, los ingredientes y la cantidad consumida. La persona responsable de preparar el alimento estuvo presente en el momento de la entrevista. En los casos en los cuales la comida consumida por un niño estaba en la escuela o en una guardería, el entrevistador visitó la escuela para obtener información detallada de las preparaciones. La calidad de los datos se controló durante todo el proceso y la entrevista se repitió en caso de inconsistencias^18^.

Se clasificaron 1.053 alimentos en una de las cuatro categorías de NOVA por los autores de este estudio. Las categorías son mutuamente excluyentes y varían según su extensión y propósito de procesamiento. Incluyen alimentos no procesados o mínimamente procesados, ingredientes culinarios procesados, alimentos procesados y alimentos ultraprocesados^1^. Los alimentos se categorizaron en uno de los 33 subgrupos. En 5,6% de los casos, no fue posible desglosar las preparaciones culinarias típicas en sus ingredientes constituyentes (por ejemplo, “Pasta para lasaña cocida”, “Arepa de choclo”, “Tamal”) y se clasificaron como alimentos mínimamente procesados en la categoría de ‘comida recién preparada’. Se calculó la ingesta de energía y nutrientes para cada alimento a nivel individual utilizando un software desarrollado por la Escuela de Nutrición y Dietética de la Universidad de Antioquia, Medellín, Colombia^18^.

Este estudio utilizó datos auto informados sobre sexo (masculino/femenino), edad (2 a 9, 10 a 19, 20 a 34, 35 a 49 y ≥ 50 años), ESE y regiones geográficas recopilados por la ENDS. El ESE de la muestra se evaluó utilizando el índice compuesto SISBEN. Este es un índice de bienestar familiar construido a partir de 24 variables en cuatro dimensiones: salud, educación, vivienda y vulnerabilidad e incluye indicadores como seguridad social, años de educación, empleo e ingreso *per capita*^19^. El nivel 1 de SISBEN indica bajo ESE, mientras que el ‘Nivel 4 y más’ indica alto ESE. El indicador del área residencial abarca categorías urbana, central y rural. Las regiones geográficas incluyen Atlántica, Oriental, Central, Pacífica, Bogotá, Orinoquia y la Amazonia.

Los datos de la ENSIN se combinaron con los datos de la ENDS para vincular el consumo de alimentos con la información demográfica a nivel individual. Las respuestas con información faltante sobre la energía total, con ingestas de energía total extrema (< 200 kcal y > 5.000 kcal)^5^ y las respuestas de las mujeres embarazadas fueron excluidas del análisis. El tamaño de muestra final incluyó 38.643 individuos. Se usaron ponderaciones de muestra en todos los análisis para tener en cuenta las probabilidades diferenciales de la selección. Los individuos se clasificaron en quintiles según la contribución dietética de los alimentos ultraprocesados (% de la ingesta total de energía). Se analizó el consumo relativo de energía (% de la ingesta total de energía) para las cuatro categorías y subgrupos de NOVA en los quintiles. Se realizaron análisis lineales de regresión bruta y ajustada con indicadores sociodemográficos y todas las categorías de NOVA y subgrupos de alimentos ultraprocesados. El nivel de significación se estableció como un alfa del 5,0% y un valor de p de 0,05. Las pruebas de diagnóstico visual para la regresión de *ordinary least squares *(OLS) sugirieron una distribución bastante normal de los errores, y desviaciones leves de lo normal en la cola superior e inferior. Los análisis de datos se realizaron en STATA 14 y el mapa fue diseñado con el programa R3,4,4.

## RESULTADOS

La edad promedio de los participantes fue de 26,5 años [desviación estándar (DE) = 0,2], 51,9% de la muestra era de sexo femenino, 29,4% tenía ESE bajo y 5,1% tenía ESE alto. Tres cuartas partes de la población residía en áreas urbanas y las regiones con mayor numero de participantes fueron Atlántico (25,3%), y Central (23,8%) (Tabla 1). Los alimentos naturales o mínimamente procesados proporcionaron el 63,3% de la energía total en la muestra total, mientras que la participación de la energía de los ingredientes culinarios procesados, los alimentos procesados y los alimentos ultraprocesados fue del 15,8%, 4,9% y 15,9%, respectivamente. Los plátanos, raíces y tubérculos fueron la subcategoría más consumida en los alimentos mínimamente procesados. El azúcar de mesa, el queso y los panes industrializados fueron los ingredientes culinarios, alimentos procesados y los alimentos ultraprocesados más consumidos, respectivamente (Tabla 2).

**Tabla 1:** Características demográficas. Encuesta nacional de la situación nutricional de la población colombiana. Colombia, 2005.

Indicador	Distribución	

	Media	Error estándar
Edad, años	26,5	0,2
	**n**	%*
**Sexo **		
Femenino	19.991	51,9
Masculino	18.527	48,1
**Grupos de edad (años)**		
2–9	7.126	18,5
10–19	8.859	23,0
20–34	10.130	26,3
35–49	7.703	20,0
≥ 50	4.699	12,2
**Posición socioeconómica**		
Nivel 1 (bajo)	10.169	29,4
Nivel 2	14.213	36,9
Nivel 3	10.977	28,5
Nivel 4 (alto)	1.964	5,1
**Área residencial**		
Urbana	28.272	73,4
Central	5.854	15,2
Rural	4.391	11,4
**Región**		
Atlántica	9.745	25,3
Oriental	6.471	16,8
Central	9.167	23,8
Pacifica	6.509	16,9
Bogotá	6.124	15,9
Orinoquia y Amazonia	423	1,1

* Porcentajes ponderados, pueden no sumar 100 debido al redondeo

Tamaño de la población con pesos de muestra = 38.519.068

**Tabla 2:** Distribución (%) de la ingesta total de energía de acuerdo con la clasificación de alimentos NOVA a través de los quintiles (Q) del porcentaje de energía proveniente de los alimentos ultraprocesados. Colombia, 2005

Grupos de Alimentos	Total 1.835 kcals	Q1 1.511 kcals	Q2 1.879 kcals	Q3 1.873 kcals	Q4 1.889 kcals	Q5 2.039 kcals
Porcentaje de calorías de UPP		0–1,5%	1,6–9,3%	9,4–17,2%	17,3–28,7%	28,8–100%

**Alimentos naturales o mínimamente procesados**	**63,3**	**76,1**	**70,7**	**64,9**	**58,4**	**44,1**
Plátanos, raíces y tubérculos (incluye harinas)	9,0	22,4	18,4	15,8	13,4	9,3
Cerales, granos (incluye harinas)	14,2	19,4	15,8	14,3	12,0	9,0
Preparaciones culinarias (listas para consumir)^a^	7,1	7,6	8,9	7,4	6,6	4,8
Leche, yogurt (naturales)	5,5	3,9	5,2	6,1	6,7	5,8
Carnes rojas	5,1	5,3	5,4	5,4	4,9	4,0
Frutas^b^	3,6	4,4	4,1	3,7	3,2	2,4
Frijoles, leguminosas, legumbres (incluye harinas)	3,5	4,2	4,0	3,7	3,3	2,0
Huevos	2,5	2,3	2,7	2,6	2,7	2,2
Carne de aves	2,2	1,9	2,2	2,4	2,5	2,0
Vegetales	1,6	1,5	1,7	1,7	1,7	1,4
Comida de mar	0,8	1,7	0,8	0,7	0,4	0,3
Jugo de fruta natural	0,3	0,2	0,2	0,3	0,3	0,3
Otros alimentos mínimamente procesados^c^	0,9	1,4	1,1	0,9	0,7	0,5
**Ingredientes culinarios procesados**	**15,8**	**19,3**	**18,3**	**16,3**	**14,1**	**10,4**
Azúcar	8,9	11,2	10,6	9,3	7,7	5,3
Aceites vegetales	6,1	6,9	6,7	6,2	5,8	4,6
Grasa animal	0,8	1,2	1,0	0,8	0,6	0,4
Otros ingredientes culinarios^c^	0,0	0,0	0,0	0,0	0,0	0,01
**Grupos 1 + 2**	**79,1**	**95,4**	**89,0**	**81,2**	**72,5**	**54,6**
**Alimentos procesados**	**4,9**	**4,4**	**5,4**	**5,6**	**5,0**	**4,4**
Quesos	1,9	2,2	2,0	2,1	1,9	1,7
Panadería (fresca sin empaquetar)	1,7	1,0	1,9	2,1	2,0	1,4
Carnes (enlatadas, ahumadas)	0,2	0,2	0,3	0,3	0,2	0,2
Frutas y verduras en conserva	0,1	0,0	0,1	0,1	0,1	0,1
Vino y cerveza	0,1	0,0	0,1	0,1	0,1	0,1
Otros alimentos procesados^c^	0,0	0,0	0,0	0,0	0,0	0,01
**Alimentos ultraprocesados**	**15,9**	**0,2**	**5,6**	**13,2**	**22,5**	**41,1**
Panes industrializados	5,0	0,0	2,1	5,3	8,2	10,3
Snacks (dulces y salados)^d^	2,5	0,0	0,7	1,8	3,2	7,5
Bebidas azucaradas^e^	2,5	0,0	0,1	2,2	3,7	6,0
Confitería (chocolate, caramelos, dulces)	1,5	0,0	0,8	1,3	2,2	3,6
Carnes procesadas	1,3	0,0	0,3	0,9	1,9	3,9
Preparaciones listas para comer “comida chatarra”^f^	0,6	0,0	0,0	0,1	0,4	2,8
Postres comerciales	0,5	0,0	0,1	0,3	0,7	1,5
Cereales de desayuno industriales	0,3	0,0	0,1	0,1	0,4	0,9
Bebidas lácteas industriales^g^	0,2	0,0	0,1	0,2	0,3	0,5
Otros alimentos ultraprocesados^c^	1,4	0,0	0,0	1,1	1,5	4,2

^a ^Incluye preparaciones culinarias dulces o saladas que no han podido ser desagregados en sus ingredientes (combinación de ingredientes principalmente del grupo 1: pastas, buñuelos, empanadas, etc).

^b ^Incluye futa en pulpa y agua de coco.

^c ^Otros: Alimentos naturales o mínimamente procesados: cocoa, carne de insectos, leche de coco, leche de soya, nueces, café, té y tofu. Ingredientes culinarios procesados: sal de mesa, pimienta, vinagre, levadura, extracto de vainilla, gelatina sin sabor. Alimentos procesados: nueces y semillas salados, endulzados o con aceite, leche condensada. Alimentos ultra-procesados: margarinas, pastillas de caldos, salsas, comidas para bebes comerciales, alcoholes destilados.

^d ^Incluye mix de mecato, galletas saladas y dulces industriales, wafers.

^e ^Incluye jugos de frutas industriales.

^f ^Incluye pizza congelada, sopas de paquete, pastas precocidas.

^g ^Incluye natillas, yogures saborizados y malteadas.

El porcentaje de contribución de las calorías totales de los alimentos naturales o mínimamente procesados, y los ingredientes culinarios y sus subgrupos, disminuyó a través de los quintiles de consumo de alimentos ultraprocesados. Las calorías de los alimentos naturales o mínimamente procesados disminuyeron un 32,0% entre el primer quintil (76,1%) y ultimo quintil (44,1%). El consumo de yogur natural aumentó entre los quintiles, mientras que el consumo de verduras no fue muy alto (1,5%) y se mantuvo sin cambios entre los quintiles. El porcentaje de calorías de todos los alimentos procesados se mantuvo relativamente sin cambios, aumentando marginalmente entre los quintiles 2 y 3, antes de volver a disminuir.

La contribución promedio de calorías de los alimentos ultraprocesados varió del 0,2% en el primer quintil al 41,1% en el ultimo quintil. Los mayores incrementos vinieron del consumo de panes industrializados, seguidos de los snacks dulces y salados, las bebidas azucaradas, las carnes procesadas y los productos de confitería. Los alimentos listos para el consumo, los postres, los cereales industrializados para el desayuno y las bebidas lácteas también aumentaron entre los quintiles, pero en un grado menor.

No hubo diferencias en el consumo de alimentos ultraprocesados entre hombres y mujeres. Se observaron diferencias significativas por edad, ESE, área de residencia y región geográfica. Los participantes de mayor edad fueron los consumidores con mayor consumo de alimentos naturales o mínimamente procesados, mientras que la categoría más joven de los participantes (de dos a nueve años) tuvo la mayor ingesta de alimentos ultraprocesados: casi el doble que la de los participantes mayores de 50 años. Los mayores consumidores de alimentos naturales o mínimamente procesados y los consumidores más bajos de alimentos ultraprocesados estuvieron en los niveles socioeconómicos más bajos, en áreas rurales o en la región Atlántica. Los habitantes urbanos, con alto ESE, que residían en la región de Bogotá tenían entre 1,5 y 1,7 más veces de ingesta calórica de alimentos ultraprocesados en comparación con sus contrapartes de bajo ESE, y sus contrapartes rurales (Figuras 1 y 2 y Tabla 3).

**Figura 1:** Distribución de la ingesta total de energía según clasificación NOVA y determinantes sociodemográficos. Colombia, 2005.

**Figura 2:** Distribución del consumo de alimentos ultraprocesados (AUP) en las regiones. Colombia, 2005.

**Tabla 3:** Variación en el consumo medio de alimentos ultraprocesados (AUP) a través de los determinantes sociodemográficos en la población. Colombia, 2005.

Factores sociodemográficos	% de energía de AUP (ES)	Beta (sin ajustar)	p	% de energía de AUP ajustado (ES)	Beta (ajustado)	p
Sexo						
Femenino	16,2 (0,3)	Referencia	-	16,2 (0,2)	Referencia	-
Masculino	15,5 (0,4)	-0,7	0,012	15,5 (0,2)	-0,6	0,007
Edad (años)						
2–9	18,5 (0,4)	Referencia	-	19,3 (0,3)	Referencia	-
10–19	18,6 (0,4)	0,1	0,678	19,3 (0,2)	-0,1	0,718
20–34	16,0 (0,4)	-2,5	< 0,001	15,4 (0,3)	-3,9	< 0,001
35–49	12,6 (0,4)	-5,9	< 0,001	12,2 (0,3)	-7,1	< 0,001
≥ 50	11,8 (05)	-6,8	< 0,001	11,4 (0,4)	-7,9	< 0,001
Posición socioeconómica						
Nivel 1 (bajo)	10,4 (0,4)	Referencia		12,7 (0,3)	Referencia	-
Nivel 2	16,5 (0,4)	6,1	< 0,001	15,8 (0,3)	3,1	< 0,001
Nivel 3	19,4 (0,4)	9,0	< 0,001	17,9 (0,3)	5,2	< 0,001
Nivel 4 (alto)	23,6 (1,1)	13,2	< 0,001	22,8 (1,0)	10,1	< 0,001
Área residencial						
Urbana	18,2 (0,3)	Referencia		17,3 (0,2)	Referencia	-
Central	9,2 (0,7)	-8,9	< 0,001	12,6 (0,5)	-4,6	< 0,001
Rural	10,4 (0,7)	-7,8	< 0,001	11,2 (0,6)	-6,1	< 0,001
Región						
Atlántica	11,4 (0,6)	Referencia	-	12.7 (0,3)	Referencia	-
Oriental	17,6 (0,8)	6,2	<0,001	18,1 (0,4)	5,4	<0,001
Central	14,8 (0,7)	3,5	<0,001	14,4 (0,4)	1,7	0,001
Pacifica	14,4 (0,6)	3,1	<0,001	14,9 (0,4)	2,2	<0,001
Bogotá	24,4 (0,6)	13,0	<0,001	21,6 (0,5)	8,9	<0,001
Orinoquia y Amazonia	17,1 (0,6)	5,7	<0,001	15,7 (0,6)	3,0	<0,001

ES = Error estándar

Las mujeres consumieron mayor cantidad de panes, snacks y productos de confitería y los hombres consumieron mayor cantidad de bebidas azucaradas y comidas listas para consumir. Los niños menores de nueve años consumieron significativamente mayor cantidad de snacks, productos de confitería, cereales procesados, bebidas lácteas y postres. Los participantes mayores de 50 años consumieron menor cantidad de productos de estos subgrupos, pero tenían el consumo más alto de pan industrializado. En las bebidas azucaradas, también se observó una tendencia diferente. Las personas entre los 20 a 34 años tuvieron las ingestas más altas (3,3% de la energía total de bebidas azucaradas) en comparación con las personas de edad avanzada (1,3%) y los niños (2,1%). De manera similar, se observó una mayor ingesta de alimentos ultraprocesados en la clase de alto ESE para la mayoría de los alimentos ultraprocesados como snacks, bebidas azucaradas, carne procesada, alimentos listos para el consumo y postres. Estos consumidores tuvieron una ingesta comparable a los de bajo ESE en pan procesado y confitería (Tabla 4).

**Tabla 4:** Porcentaje de energía proveniente de alimentos ultraprocesados (AUP) y sus subgrupos según determinantes sociodemográficos. Colombia, 2005.

Indicadores	Todos los AUP	Panes Industrializados	Snacks (dulces y salados)^b^	Bebidas azucaradas^c^	Confitería	Carnes	Comida chatarra^d^	Postres comerciales	Cereales	Bebidas Lácteas^e^	Otros AUP^f^
Sexo											
Femenino	16,2 (0,,2)	5,2 (0,1)	2,8 (0,1)	2,2 (0,1)	1,7 (0,1)	1,4 (0,1)	0,6 (0,0)	0,6 (0,0)	0,4 (0,0)	0,2 (0,0)	1,2 (0,1)
Masculino	15,5 (0,,2)	4,8 (0,1)	2,2 (0,1)	2,8 (0,1)	1,3 (0,0)	1,3 (0,1)^a^	0,7 (0,1)	0,4 (0,0)	0,2 (0,0)	0,2 (0,0)^ a^	1,6 (0,1)
Edad (años)											
2 – 9	19,3 (0,3)	5,2 (0,1)	4,5 (0,1)	2,1 (0,1)	2,3 (0,1)	1,6 (0,1)	0,5 (0,1)	0,9 (0,1)	0,6 (0,0)	0,5 (0,0)	0,9 (0,1)
10 – 19	19,3 (0,2)	5,4 (0,1)^ a^	3,8 (0,1)	2,8 (0,1)	2,1 (0,1)	1,7 (0,1)^ a^	0,9 (0,1)	0,7 (0,0)	0,3 (0,0)	0,2 (0,0)	1,2 (0,1)
20 – 34	15,4 (0,3)	4,6 (0,2)	1,8 (0,1)	3,3 (0,1)	1,1 (0,1)	1,4 (0,1)^ a^	0,6 (0,1)^ a^	0,3 (0,0)	0,2 (0,0)	0,1 (0,0)	1,9 (0,2)
35 – 49	12,2 (0,3)	4,7 (0,2)	1,1 (0,1)	2,1 (0,1)^ a^	1,0 (0,1)	1,0 (0,1)	0,3 (0,1)	0,3 (0,1)	0,1 (0,0)	0,1 (0,0)	1,4 (0,1)
≥ 50	11,4 (0,4)	5,5 (0,3)^ a^	0,9 (0,1)	1,3 (0,1)	1,0 (0,1)	0,8 (0,1)	0,4 (0,1)^ a^	0,2 (0,1)	0,1 (0,0)	0,1 (0,0)	1,3 (0,2)^ a^
Posición socioeconómica											
Nivel 1 (bajo)	12,7 (0,3)	4,4 (0,2)	1,9 (0,1)	1,8 (0,1)	1,4 (0,1)	1,0 (0,1)	0,3 (0,0)	0,3 (0,0)	0,1 (0,0)	0,1 (0,0)	1,3 (0,1)
Nivel 2	15,8 (0,3)	5,4 (0,1)	2,3 (0,1)	2,5 (0,1)	1,6 (0,1)	1,3 (0,1)^ a^	0,6 (0,0)	0,5 (0,0)	0,3 (0,0)	0,2 (0,0)	1,3 (0,1)^ a^
Nivel 3	17,9 (0,3)	5,2 (0,1)	3,1 (0,1)	2,9 (0,1)	1,5 (0,1)^ a^	1,6 (0,1)	0,8 (0,1)	0,6 (0,1)	0,4 (0,1)	0,3 (0,0)	1,5 (0,1)^ a^
Nivel 4 (alto)	22,8 (1,0)	5,2 (0,4)^ a^	3,8 (0,4)	3,7 (0,3)	1,4 (0,2)^ a^	2,3 (0,4)	1,8 (0,3)	1,0 (0,1)	0,7 (0,1)	0,6 (0,1)	2,2 (0,4)
Área residencial											
Urbana	17,3 (0,2)	5,4 (0,1)	2,6 (0,1)	2,8 (0,1)	1,5 (0,1)	1,5 (0,1)	0,7 (0,0)	0,6 (0,0)	0,3 (0,0)	0,2 (0,0)	1,4 (0,1)
Central	12,6 (0,5)	4,2 (0,2)	2,3 (0,2)	1,8 (0,1)	1,5 (0,1)^ a^	0,9 (0,1)	0,4 (0,0)	0,4 (0,1)^ a^	0,2 (0,0)	0,1 (0,0)	0,8 (0,1)
Rural	11,2 (0,6)	3,5 (0,3)	1,5 (0,1)	1,4 (0,2)	1,3 (0,2)^ a^	0,7 (0,2)	0,2 (0,1)	0,3 (0,1)^ a^	0,1 (0,0)	0,1 (0,0)	2,1 (0,4)^ a^
Región											
Atlántica	12,7 (0,3)	3,5 (0,1)	2,2 (0,1)	2,4 (0,1)	0,5 (0,0)	1,3 (0,1)	0,5 (0,0)	0,3 (0,0)	0,2 (0,0)	0,2 (0,0)	1,4 (0,1)
Oriental	18,1 (0,4)	6,3 (0,3)	2,4 (0,1)^ a^	3,0 (0,1)	2,1 (0,1)	1,1 (0,1)^ a^	0,6 (0,1)^ a^	0,6 (0,1)	0,4 (0,1)	0,1 (0,0)^ a^	1,4 (0,1)^ a^
Central	14,4 (0,4)	3,1 (0,2)^ a^	2,6 (0,1)	2,3 (0,1)^ a^	1,3 (0,1)	1,7 (0,1)	0,7 (0,1)	0,6 (0,1)	0,3 (0,0)	0,3 (0,0)	1,5 (0,2)^ a^
Pacifica	14,9 (0,4)	5,5 (0,2)	2,6 (0,1)	2,1 (0,1)	0,9 (0,1)	1,4 (0,1)^ a^	0,4 (0,1)^ a^	0,4 (0,0)	0,2 (0,0)^ a^	0,2 (0,0)^ a^	1,1 (0,1)^ a^
Bogotá	21,6 (0,5)	8,2 (0,3)	2,8 (0,2)	2,6 (0,2)^ a^	3,3 (0,2)	1,0 (0,1)^ a^	0,9 (0,1)	0,7 (0,1)	0,4 (0,1)	0,2 (0,0)^ a^	1,4 (0,2)^ a^
Orinoquia y Amazonia	15,7 (0,6)	4,9 (0,3)	2,5 (0,2)^ a^	3,4 (0,2)	1,7 (0,2)	1,1 (0,1)^ a^	0,6 (0,1)^ a^	0,4 (0,1)^ a^	0,3 (0,0)	0,1 (0,0)^ a^	0,7 (0,1)

^a^ No hay diferencias significativas comparado con el grupo referencia en cada variable (en sexo: mujeres, edad: 2-9 años, región: Atlántica, Área: urbana, Posición socioeconómica: Baja, después de ajustar para todas las variables sociodemográficas.

^b ^Incluye mezcla de mecato, galletas saladas y dulces industriales, wafers.

^c ^Incluye jugos de frutas industriales.

^d ^Incluye pizza congelada, sopas de paquete, pastas precocidas.

^e ^Incluye natillas, yogures saborizados y malteadas.

^f ^Incluye margarinas, pastillas de caldos, salsas, comidas para bebes comerciales, alcoholes destilados.

Los participantes que residían en áreas urbanas consumieron mayor cantidad de alimentos ultraprocesados. Sin embargo, su consumo no fue significativamente diferente comparado con los residentes rurales respecto a los dulces de confitería. Se observaron tendencias similares entre los residentes de la región de Bogotá que consumieron significativamente mayor cantidad de pan industrializado, confitería, aperitivos, alimentos listos para el consumo y postres que otras regiones. Sin embargo, el consumo de carnes procesadas fue el más bajo. Los residentes de Orinoquia y la Amazonia tuvieron la mayor ingesta de bebidas azucaradas, mientras que los de la región Central superaron el consumo de carne procesada. Casi no se observaron diferencias entre regiones geográficas para la ingesta de bebidas lácteas.

## DISCUSIÓN

Los datos mostraron una amplia gama en los patrones de consumo de alimentos ultraprocesados en Colombia según factores sociodemográficos. Aquellos individuos con el consumo más bajo de alimentos ultraprocesados (primer quintil de consumo de alimentos ultraprocesados, < 1% del consumo total de energía), obtuvieron más del 95,0% de su consumo de energía de alimentos naturales o mínimamente procesados e ingredientes culinarios. Por otra parte, los individuos con mayor consumo de alimentos ultraprocesados desplazan casi la mitad de las calorías provenientes de los alimentos naturales o mínimamente procesados - obtuvieron el 41% de su consumo de energía de los alimentos ultraprocesados y el 44% de los alimentos naturales o mínimamente procesados. El pan industrializado fue el mayor contribuyente a la energía total proveniente de alimentos ultraprocesados. Los adolescentes y los residentes urbanos fueron los mayores consumidores de alimentos ultraprocesados, especialmente snacks, cereales procesados, bebidas lácteas y comida chatarra. Los hombres consumían mayor cantidad de bebidas azucaradas y comidas listas para comer.

A medida que el consumo total de alimentos ultraprocesados aumentó, el pan industrializado, los aperitivos y las bebidas azucaradas proporcionaron la mayor cantidad de energía, desplazando el consumo de plátanos, raíces y tubérculos, y cereales provenientes del grupo de los alimentos naturales o mínimamente procesados. Los subgrupos de alimentos ultraprocesados que contribuyen a la mayoría de las calorías varían en otros países de América del Sur. Los postres, las galletas, pasteles y bebidas azucaradas son los principales contribuyentes a la energía en México y Chile^20,16^. Los postres, las comidas rápidas y las bebidas azucaradas son los principales contribuyentes en Brasil^4^. La confitería, las carnes procesadas y las comidas listas para el consumo también aumentaron en Colombia, pero en menor medida. En general, el consumo de alimentos ultraprocesados en Colombia sigue relativamente bajo, pero con un enorme potencial de crecimiento del consumo en todos los subgrupos según datos de tendencia de compra en los últimos años^15^.

En Colombia, los residentes urbanos, menores de 19 años de la capital Bogotá y de alto ESE son los principales consumidores de alimentos ultraprocesados. De estos, los niños son el grupo más vulnerable para el consumo de estos productos. Esto es particularmente preocupante considerando las consecuencias a largo plazo para la salud de la ingesta de alimentos ultraprocesados^8,9^. Un patrón dietético caracterizado por el consumo de subgrupos de alimentos ultraprocesados como hamburguesas y *hot dogs* (“perro caliente”) se asoció con mayor sobrepeso entre los niños colombianos de 5 a 12 años, particularmente aquellos en el alto ESE^21^. Es probable que los residentes rurales y adultos mayores tengan prácticas de cocina y alimentación más tradicionales y patrones dietéticos más estables. Estos grupos también pueden ser más resistentes a las prácticas de comercialización que atraen a la generación más joven, con patrones dietéticos menos estables, y por lo tanto más propensos a experimentar con estos productos.

Aspectos como la exposición, la disponibilidad, el acceso, y la penetración de estos alimentos ultraprocesados en el entorno alimentario de Colombia también pueden explicar el más alto consumo de estos productos en las zonas urbanas, de alto ESE y en las grandes ciudades (principalmente en la capital, Bogotá). Es probable que estos segmentos tengan una mayor exposición al marketing, mayor poder adquisitivo para el acceso a estos productos y un paladar más amplio para adaptarse a los gustos nuevos. Los colombianos que residen en las zonas rurales, en las regiones del Atlántico, Central y el Pacífico o los del bajo ESE, están protegidos de la exposición a estos alimentos ultraprocesados, conservando las preparaciones culinarias tradicionales. Eso se debe probablemente a la menor exposición a los determinantes comerciales y marketing de estos productos y a los bajos ingresos disponibles.

El patrón colombiano de consumo de alimentos ultraprocesados entre los niños y adolescentes refleja el patrón global: el Reino Unido, Estados Unidos, Canadá, Chile, Francia y México muestran un mayor consumo de alimentos ultraprocesados dentro de estos segmentos de edad de la sociedad^7,16,20^. Por otro lado, el consumo no es tan consistente para el ESE en México y Brasil, así como en Colombia, los más ricos y educados consumen más alimentos ultraprocesados, con una dosis-respuesta vista al aumento según ESE^20,24^. Chile también tuvo un mayor consumo para aquellos con mayores ingresos, pero no se encontraron diferencias por educación^16^. Sin embargo, el consumo de alimentos ultraprocesados fue mayor entre los que tenían educación y el nivel de ingresos más bajo el Reino Unido, Francia y los Estados Unidos. Estos resultados apuntan a una distinción en los patrones de consumo según la etapa de transición nutricional de cada país. En los países en desarrollo en la etapa intermedia (patrones 3 y 4) de transición nutricional, la compra y el consumo de alimentos ultraprocesados fueron impulsados por individuos con altos niveles de ingresos. Estos países se alejan de la ingesta de alimentos amiláceos (maíz, trigo, arroz, avena, etc.) y de las deficiencias proteico-energéticas hacia las enfermedades no transmisibles^25^. Lo contrario se observó para los países desarrollados que se acercan al final (patrón 4) de transición nutricional. En estos países, aunque el consumo fue relativamente bajo entre los más ricos, el porcentaje de energía diaria de los alimentos ultraprocesados es superior al 50,0%^25^.

Pocos estudios han examinado las diferencias dentro de los subgrupos de alimentos ultraprocesados. En Noruega, los hombres fueron más propensos a ser clasificados como grandes consumidores de productos de cena ultraprocesados y comidas rápidas, pero no de aperitivos y bebidas azucaradas, en comparación con las mujeres^26^. Aquellos con un alto nivel de educación tenían menor probabilidad de consumir alimentos ultraprocesados en la cena y aperitivos y bebidas azucaradas durante el día en comparación con aquellos con un bajo nivel de educación. Estos resultados fueron diferentes a los que se vieron en este estudio y probablemente se explican por la etapa posterior de la transición nutricional (patrón 4) en Noruega. Entre los niños, hay cierta consistencia en los tipos de alimentos ultraprocesados consumidos. En análisis de componentes principales del consumo de alimentos en 12 países muestran que los alimentos ultraprocesados como comidas rápidas, helados, frituras, papas fritas, pasteles y bebidas azucaradas caracterizan con mayor fuerza los patrones alimentarios no saludables entre los niños de nueve a 11 años^27^. Los datos recientes de Colombia sobre la frecuencia del consumo de bebidas azucaradas muestran que el 85,3% de los niños entre 5 y 17 años consumen estas bebidas 0,71 veces/día^28^. La contribución porcentual de las bebidas azucaradas a la energía diaria no estaba disponible, pero las tendencias de la frecuencia apuntan a un patrón de consumo muy presente y preocupante entre los niños y adolescentes.

Es necesaria una regulación para proteger el estado nutricional y el bienestar, especialmente de los niños y adolescentes^12^. Las estrategias pueden incluir: (i) la prohibición de la venta de alimentos ultraprocesados en escuelas y patios de recreo, (ii) una mejor diseminación de herramientas de promoción de la salud, tal como las guías dietéticas basadas en alimentos que consideren la extensión y el propósito del procesamiento implementadas en Brasil^29^ y Uruguay^30^, (iii) un etiquetado de advertencia e iniciativas impositivas apropiadas para alentar opciones más saludables en los puntos de compra como se ha realizado en Chile^10^.

Este estudio tiene sus limitaciones. Toda la información dietética se recopiló mediante recordatorio dietético de 24 horas. Más allá del sesgo de la memoria, los participantes pueden optar por retener información sobre ciertos productos alimenticios que se consideran socialmente indeseables, tanto en términos de categorías de productos como de cantidades consumidas. Este único punto de información dietética puede no capturar la dieta habitual de los participantes y, por lo tanto, ser menos representativa de su ingesta. Aunque los análisis controlaron varias variables sociodemográficas, la confusión residual es difícil de eliminar. Sin embargo, la naturaleza probabilística de la muestra estudiada, la representatividad nacional de la población colombiana usando la última encuesta nacional con recordatorio dietético de 24 horas disponible, y la estandarización de la recolección de datos dietéticos son algunas de las fortalezas de este estudio. Es probable que el consumo de alimentos ultraprocesados en Colombia haya cambiado desde la publicación de estos datos. Este estudio proporciona niveles de referencia de ingesta y podría ser una línea de base que podría compararse con evaluaciones dietéticas más recientes, cuando estén disponibles, para captar las tendencias en el consumo de alimentos ultraprocesados en Colombia.

## CONCLUSIÓN

Este estudio caracteriza los alimentos ultraprocesados que se consumen con mayor frecuencia y que se pueden considerar como los puntos de entrada de los alimentos ultraprocesados dentro de la dieta colombiana. Se exploran las diferencias sociodemográficas para identificar los segmentos de la población que pueden considerarse los más vulnerables al consumo de estos alimentos. El pan industrializado es el alimento ultraprocesado más fácilmente asimilable en la dieta tradicional, junto con los snacks y las bebidas azucaradas. Los niños y adolescentes se adaptan más fácilmente a estos productos y, por lo tanto, son más vulnerables a sus efectos deletéreos sobre la salud. En general, el consumo de alimentos ultraprocesados no es tan alto en Colombia como en otros países, pero con una mayor probabilidad al aumento. Estos datos también estimulan una revisión de la política actual de promoción y prevención de la salud y a los esfuerzos regulatorios en Colombia relacionados con la promoción y la venta de estos productos, particularmente en poblaciones vulnerables como los niños y adolescentes.
